# Address Introductory to the Twenty-Second Annual Course of Lectures in Rush Medical College

**Published:** 1864-11

**Authors:** De Laskie Miller

**Affiliations:** Professor of Obstetrics and Diseases of Women and Children


### CHICAGO MEDICAL JOURNAL.

VOL. XXI.] NOVEMBER, 1864. [No. 11.

### ORIGINAL COMMUNICATIONS.

### ADDRESS INTRODUCTORY TO THE TWENTY-SECOND ANNUAL COURSE OF LECTURES IN RUSH MEDICAL COLLEGE.

By DE LASKIE MILLER, M. D.,

*Professor of Obstetrics and Diseases of Women and Children.*

[Published by Request of the Class.]

*Young Gentlemen, Members of the Class:—*

The occasion which has convened us this evening is one of no ordinary interest—to you, in view of the labors which you are undertaking, and in the associations which you are about to form—many of which, we trust, will go with you through life, bearing with them pleasing memories of the present; to your instructors in the new relations now established, by which, to some extent, we assume the responsibility of the proper improvement of your time while these relations exist. These circumstances ever render the first meeting of a medical class interesting.

It is not, however, the interest of idle curiosity, nor the novel circumstances which surround you on first entering a medical college, which shall suggest my theme for the short time that I may detain you this evening. I remember that you are students—that you are to become medical scholars—thus I am admonished that you are here for a purpose; you would be qualified to act some honorable and useful part in the world's great drama. I speak of the progress of the race—of the conflict of mind with mind—by which the arts and sciences are carried forward to increasing usefulness and perfection, and by which our civilization is advanced and rendered practical in elevating and blessing society. To live, at the present time, involves great responsibility; life is active, earnest, real; it has become intensified in a thousand ways, so that the individual owes it to himself and to society, to put forth his best efforts in the great cause of humanity; and this he must do or consent to be distanced in the race of life; for in the on-rushing car of human progress, no standing room is left to idlers—no sleeping room for dreamers.

All who are only tolerably familiar with the modern achievements of medical science, will unhesitatingly concede that this general statement of the progress of events is not less true when applied specifically to the profession which you have chosen. In entering upon its labors and responsibilities, you will do well to emulate the example of the many worthies who have ascended to pre-eminence in our ranks by the awards of labor and of merit.

You have but entered the unbounded and ever-expanding field of responsibility, of usefulness, and of pleasure, whose outskirts you may never reach, but whose development, in a great measure, is in your own hands. *Mark well*, therefore, your beginnings, with each step of your progress in the formation of your mental, moral and professional character, in each of which, be assured that, industry, enlightened reason and purity of life are essential to success.

Possessing and exercising these, the ultimate result of your exertions is no longer problematical. On this foundation you may rear a superstructure that shall command the admiration of all men for its symmetry, strength and utility, the materials for which are already offered to your hands, in the physical sciences, in the experience and contributions of ages. As if you were passing along some vast apartment filled with the most exquisite productions of genius and art, you have but to reach forth and select from the vastness and variety around you the material suited to your purpose. Your embarrassment will not arise from, where to find, but, what to choose. Even the most gifted, who have enjoyed the highest advantages, are forced to confess that they stand on the shore of the great ocean of knowledge, collecting a few pebbles and shells, while its depths have not been sounded nor its boundaries reached. A single branch of learning offers labor sufficient to occupy the longest life. This is especially true of the science and art of healing.

It is not my purpose to pronounce a panegyric on the science of medicine, but rather turn to topics which, if less inviting, still possess a practical interest for you at the present time.

You visit this city for a common purpose, many of you have left, in the distance, pleasant homes and dear friends, that you may avail yourselves of the advantages which we offer, to prepare for the arduous duties of the physician. I do no injustice to any when I assert that skill in the art of healing is not insured by the advantages within the reach of the individual.

The example is not solitary, of a student, surrounded by all the aids to mental improvement, added to the virtuous habit of a reasonable amount of industry, which, by being injudiciously applied, yield but a record of wasted years, and a mind void of understanding. A medical education is not to be acquired, as the air we breathe, by inspiration, nor does the mind become stored with useful knowledge as the sponge is filled with water. If any have cherished this vain deceit, the ordeal of experience will surely and sadly prove, that when weighed in the balance such will be found wanting. I would disabuse the mind of the false notion, that nothing more is necessary for you, but to sit passively on those seats, and inhale the atmosphere of the medical college to improve the mind and develop the intellect.

Are you sure that you comprehend the import of the terms, when we speak of the improvement of the mind and intellectual development? or the means by which these may be best attained? Too often the mind is regarded as some great indefinable, incomprehensible entity; requiring for its growth and development only to be surrounded by folios, quartos, and octavos; a greater fallacy could hardly be entertained.

The elementary constituents of all knowledge consist in a three-fold operation, the objective, the subjective, and impersonal. Objective ideas arise in external facts, the subjective in registered impressions, the impersonal issue of pure reason, as, for example, the abstract truths of geometry, and are, therefore, to be attributed to the essential nature of the intellect.* On the faculties of perception, memory and comparison depend our ability to judge of facts and things, and hence the avenues to the understanding are mainly through the special senses.

The necessity to the physician of possessing and preserving the senses intact thus appears evident, for how shall he who is deprived of vision ever appreciate the signs of disease which are presented to the eye alone? He cannot discriminate the different eruptive diseases, or detect the line on the gums which indicates lead poisoning, nor estimate the value of the ever-varying expression of the countenance in disease. It is related of a late eminent surgeon that, so thoroughly had he studied the physiognomy in disease, he required little else to diagnose the affection of his patient. Being called in consultation in an obscure case, he observed the countenance of the patient but for a moment, and immediately enquired, "Where is the abscess?" Subsequent examination revealed a large

* Draper's Physiology.

collection of pus which had been unsuspected. Nor can the deaf man ever avail himself of the nice distinctions of sound, which the march of science has made to reveal the secrets of the breast. Nor can he whose fingers are cased in horn ever cultivate the sense of tact so necessary to the physician and surgeon. In vain would he attempt the acuteness of Thabit Abrahim, of whom it is related that, by placing his finger on the artery for an instant, he could tell the seat and nature of the disease, and more wonderful still, whether his patient had dined on beef or mutton, or drank for breakfast coffee from Hodeida or coffee from Mocha. To educate the senses for far less marvelous feats than those told of the Arabian leech, requires a natural talent, for if nature opposes, all else is vain. In the language attributed to Hippocrates, "It is the business of the physician to know, in the first place, things similar and things dissimilar; those connected with things most important, most easily known, and in anywise known; which are to be seen, touched, and heard; which are to be perceived in the sight, and the hearing, and the touch, by olfaction and taste, and by the understanding; which are to be known by all the means we know other things."

Possessing these requisites, the means for cultivating the intellect are various. On account of its general applicability, *reading* holds the first place. It brings to the being whose existence is measured by a brief hour, the accumulated learning of centuries; to him of feeble intellect the achievements of the greatest minds. Here we may sail on the deep sea of accumulated facts and view the silent world of passing events. Here we may select our associates in ancient or modern character. Here we may converse with the ancient seer, philosopher, poet, historian. Thus in books we are to seek patterns in learning, in refinement and taste, in reasoning, and in ethics. By common acceptation reading has come to be used as synonymous with study, so that the student is said to be reading medicine, law or theology.

To study implies the necessity of reading, and many, therefore, imagine that all readers are students. Each of you will appreciate the magnitude of the mistake by a little reflection. Have you not, at some time, when endeavoring to fix the attention on the description of the *os sphenoides*, or some other equally interesting and entertaining subject, been annoyed when near the bottom of the page, to find that the mind retained but a dim picture of the outlines of an osseous structure devoid of form or comeliness? The eye has followed the lines mechanically, while the mind has been reveling in scenes far away, with loved ones at home, or possibly the beautiful features, the ruby lip and lustrous eye of one left behind you, has caused the mind thus to play truant.

Need I tell any of you that time thus occupied is worse than thrown away. You can acquire no available knowledge in this manner. It is like reading Euclid's Elements, as the young man did, who had a happy genius of making everything easy to himself; he boasted of reading the whole of Euclid in a small part of one afternoon, and found it a pleasant entertainment. "But did you master all the demonstrations, and solve all the problems as you went?" inquired a gentleman. "I suppose," said the genius, "you mean the A's and B's and C's, and 1's and 2's, and the pictures of scratches and scrawls. Oh, no, I skipped all them." Alas, how many such readers there are in the world!

We are told by certain metaphysicians that impressions once made upon the mind are never effaced, and remarkable instances are cited to confirm the statement, such as the following: An individual in the excitement or delirium of fever may relate facts that in health had long since passed from the memory, or he may speak a language which he had only heard incidentally; and to strengthen belief in the stability of impressions, curious philosophical experiments are described. If upon the polished surface of a piece of metal a wafer be laid, and then breathed upon, and after the moisture has disappeared, the wafer being thrown off, though the most critical inspection does not reveal the slightest trace of any form, breathe upon it again and a spectral figure of the wafer comes distinctly into view. And this may be repeated again and again, even after the lapse of many months. If a piece of paper on which a key or other object has been lying, be carried into the sunshine and then be instantaneously viewed in the dark, the key having been removed, a fading spectre of the key will be seen, and the figure may be made to reappear afterwards by laying the paper on the surface of a heated metal plate. It is assumed that every shadow that falls upon a wall on any of our crowded thoroughfares, is indelible, and requires only the application of some unknown process to render it cognisable by the sense of vision. Let this be true, and further, concede that these experiments are applicable to mental impressions, still, that student errs greatly who relies on knowledge held by so feeble a tenure as to be developed only in the delirium of fever, or with learning that may be rendered available only under peculiar circumstances. He should possess knowledge that is completely under the control of the will, that may be commanded under every emergency, else he may call for it in the hour of need, as he would

> "Call spirits from the vasty deep,"

but it will not come.

There are those who would tell you that when the mind wanders from the subject, to defer every attempt to study for the time, and divert yourselves in any harmless amusement. I advise quite the contrary, when you fail to comprehend the full meaning of any page or paragraph, be it from any cause, commence and read it again, and repeat this if necessary, till you master it. I know the practice is hard and exceedingly annoying, but it can be done. And in this way alone can you subject the faculties of the mind and really cultivate the intellect.

It is my duty, in passing, to admonish you that it is not a matter of indifference what books you read. All books are not equally valuable, and it is seldom at the present day that we can justly estimate the value of a book, as they did when

Rome was in the zenith of her glory, at twice and even three times its weight in gold. And more than one reason might be cited to explain the depreciation in this kind of stock. The following curious statistics, taken from a book published in 1822, explains the want of value in a large proportion of books. "There are 1,000 books published per annum in G. B., on 600 of which there is a commercial loss, on 200 no gain, on 100 a trifling gain, and only on 100 any considerable profit. 750 are forgotten within the year, another 100 in two years, and another 100 in three years, not more than 50 survive seven years, and scarcely 20 are thought of after twenty years. Of the 50,000 books published in the 17th century, not 50 are now in estimation, and of the 80,000 published in the 18th century not more than 300 are considered worth reprinting, and not more than 500 are sought after in 1822. Since the first writings, 1400, B. C., *i. e.*, in thirty-two centuries, only about 500 works of all nations have sustained themselves against the devouring influence of time." I regret to be compelled to say that while the multiplication of books during the last forty years has increased, *pari passu*, with the advance of the Arts and Sciences, the ratio of valuable original books is not greater than in early times. Then how worse than useless to consume your time over a book, the very existence of which will have passed from the memory of men in a decade.

You should select the best book upon the subject you are investigating, and make yourselves perfectly familiar with it. Then you may read as many other works upon the same subject as you have time or inclination to. But you cannot rely upon your own judgment in selecting the first, you must then be guided by others in whose intelligence and judgment you can confide. You will appreciate the importance of this judicious selection of books more and more as you become conversant with the sciences, for you will be surprised to find how few books contain the sum of human knowledge, in any department of learning. I may then commend to you the advice given by the younger Pliny, "To read much but not to read many books."

You will not, I am sure, infer from anything that I have said, that the rapidity with which you pass through a book is any measure of your advancement in knowledge; for though you might finish a volume in a day, you would most likely come to the same conclusion reached by a medical student whom I knew some years since, who relinquished the study of medicine, "because he could read only a hundred pages a day." Though by no means an easy task, the habit of reading thoughtfully and with reflection, is as essential as reading the best works. As well might we expect the body to gain strength by constantly eating, without the power of assimilation, as the mind to be improved without thought; the injunction "to read, mark, learn, and inwardly digest," is peculiarly applicable to the medical student.

*Lectures* have long been one of the main appliances in teaching, first, as a necessity, when the scarcity of books made it difficult to obtain them, and now, when books abound, the lecture is found to be the most efficient means of instruction. The hour for the lecture commands attendance; the presence of the living teacher, his voice and occasional question ensure attention; the illustrations by diagrams, drawings, models and preparations, the experiments in chemistry, the demonstrations in anatomy, and the vivisections in physiology, as practiced in this college, addressed to the eye, make a lasting impression, so that none can go away without some addition to their stock of learning. The active attention is not less important in the lecture-room than in reading, for the page may be re-read if not comprehended at first, but by inattention, only for a moment, an important fact or principle may be ennunciated, and escape the dull ear beyond recovery. Every teacher may with propriety address his class in the language of the Roman patriot, "Hear me for my cause, and be silent that you may hear. Censure me in your wisdom, and awake your senses that you may the better judge." Lectures, demonstrations, and experiments, however, will not supercede entirely the necessity of reading during the term. For it may happen that some topic may not be fully elaborated in the lecture; or if so, it would not be surprising if, in a large class, an idea should be misapprehended, or not entirely comprehended, by some one. Then when you review the labors of the day, as you should do each night, in case of doubt upon any point, you should immediately turn to your text-book to supply the deficiency. I would not ask you to read the entire subject of all the lectures as they are given each day, but read to correct mistakes and refresh the memory when necessary.

Should the student take notes in the lecture-room? From considerable observation, I am certain that much of the labor devoted to taking notes of lectures is unprofitable; and this is most likely to be the case with students attending their first course. From their want of familiarity with the subject, they cannot readily discriminate between the non essential and the important, but in subsequent courses they should be prepared to appreciate and note down the facts, principles and conclusions announced by the teacher, and this may be of real service. Then during the first course, I believe the student will profit most by listening attentively to the lectures, and in the evening he may take notes of the lectures in the order in which they were given during the day. This course will exercise the memory, and fix instruction in the mind. This exercise of the memory will surprise most of you in its results, it is wonderful how soon you will acquire the ability to remember not only the ideas but also much of the language of the lecture. It is true you may not at first vie with the Emperor Claudius, who retained in memory all of Homer, Sallust, Demosthenes, Avicen, and Aristotle's Metaphysics. It is said of Tully and Seneca, that they never heard anything material, but it was imprinted on their memory. You need not fear the regret said to have been expressed by Themistocles, in the following language, "I had rather be taught the art of forgetfulness, for I remember those things I would not, and I can not forget those things I would."

Students who commence their medical studies by a course of lectures are many times in doubt whether they should attend the entire course or devote special attention to particular branches. For two reasons I think they should attend all the lectures: First, as a rule, those who attempt most accomplish most, and the student who listens to every lecture will, at the conclusion of the course, have a better knowledge of medical science than he who limits himself to a partial course; and second, all recognized Medical Colleges require as a condition for graduation that the candidate shall have attended two full courses of lectures. Many unforeseen circumstances may interfere to prevent the student from attending two subsequent courses, when the partial course might be filled up, and thus he may be disappointed in obtaining the degree when he greatly desires it. And even should nothing prevent the student from attending three courses of lectures, which is by all means desirable when practicable, the course we advise has these additional advantages: By attending three complete courses, a better knowledge of medical science would be almost necessarily acquired than by attending three partial courses, while the expense of tuition would not be increased, for, after attending two complete terms, his right to attend subsequently, free, becomes perpetual.

By the means enumerated, you may become learned in the science of medicine; you may be enabled to talk fluently, and write graphically of disease; and still fall short of being skillful physicians. Something more is necessary to render your knowledge available in the sick room. You must acquire a certain practical tact, to remove the embarrassments that would otherwise meet you at every turn.

The foundation of medical experience is observation of disease, and the requisites of successful observation are minuteness and accuracy. Thus you see the importance of clinical study. In books disease is delineated in bold outlines, which may lead you to believe that all doubt and uncertainty are swept away; but clinical experience will very soon undeceive you, for in practice you will seldom find these distinctive characteristics strongly pronounced; the idiosyncrasis of the constitution, the complications of distant organs, through what is called sympathy, so mask the morbid state of the suffering organ, that you will feel no little doubt in determining which set of symptoms are accidental, which are diagnostic. From this difficulty you will be relieved by your professor of clinical medicine, who will make the examination of patients in your presence, and eliminate the false from the true, the important from the accidental symptoms, and thus enable you to see at a glance the seat and nature of the disease.

The first step to successful clinical study is to select a representative case. In this, students not unfrequently make a mistake by selecting some remarkable and unusual case for observation and study, and one it may be, too, not amenable to treatment. Whereas much more profit would be gained by devoting the same time to some case of frequent occurrence. Then note carefully every particular relating to it. Let the symptoms represent the letters; combine these to form words; then arrange these words into a sentence, which shall read to your mind the history, the nature and the treatment of the disease. It is necessary to be thus minute and accurate in every case. It should become habitual. And you will at length acquire a tact of reading the case at a glance, as you would the printed page, without fixing the attention especially on the elements—the letters—of which it is made up.

No attainment is more important to the physician than well cultivated habits of observation. To observe is to examine; it implies intention. We may notice an object because it is cast before us; but observe it only when we make it a source of our reasoning. We may, therefore, observe by means of the senses, or by the process of induction. The physician must observe minutely, in order to distinguish accurately. He may know the character of the disease, but to distinguish its degree of progress often requires great discriminating powers and careful observation. The careful observer does not merely notice all the minute points of a case, but reflects upon them, associates them together. He gleans from every circumstance the source of his reasoning. He considers the history of the case—the expressions of the countenance—all the surroundings. Thus as the boy reads the page at a glance, so the educated physician is finally enabled to read the true condition of his patient. One case elaborated thus will be worth more to you than many cases examined cursorily and without order.

Your improvement will depend not more upon the number of cases at your disposal than upon the use you make of them. What you undertake in this department perform as you would anything else, to the best of your ability, minutely, accurately.

In your first attempts thus to study disease, you will, as others have before you, meet with difficulties. Should these embarrassments discourage you, and lead you to conclude that the practice of medicine is at best a conjectural art, I ask you to remember that in the early dawn of society the indications for cure were drawn from sources as insubstantial "as the baseless fabric of a vision," from dreams, from incantations, charms, and the inexplicable utterances of oracles. Now, practice is based on a knowledge of pathology that draws its interpretations from the science of physiology. How minute, how precise, how connected and definite now. How loose, indefinite, uncertain, how vague and unconnected then.

I anticipate the time when the explanations of physiology shall leave no point in the complex functions of our organisms unexplained; and on this perfected knowledge, the practice of medicine should vie in accuracy with the mathematical sciences of this day. In these achievements is my hope of the utter extinction of quackery from the pale of society. Let us then be thankful for the blessings of the present, and hopeful in the bright promise of the future.

The means which we place before you, during the course, will be ample to aid you in becoming scientific and skillful physicians. If proof of this were needed, we have but to refer to the long list of those who have preceded you from this institution. And it is with no ordinary feelings of gratification that I am enabled in this public manner to refer to the character and success of more than seven hundred alumni of this College, who have attained the most responsible positions, in both civil and military practice, and have filled them with credit to themselves and honor to their *alma mater*. *Probatum est*, thus do we prove that the facilities and the arrangement of the course which we commend are all that is essential to conduct the student to eminence in the profession. We do not claim to have exclusive control of any great discovery in the art of teaching which promises to relieve the pupil from the personal labor of studious application, or to lead him by new and enchanting paths to the object of his aspirations, for such promises ever prove fallacious; such paths, though flowery, too often prove devious, and lead to disappointment.

In selecting the profession of medicine as the vocation for your life's labor, the question of importance which should be well considered is—Have you chosen well? If to live for yourselves, to amass a fortune, to enjoy luxurious ease, and as you appear in public to be designated by the splendor of your equipage, be your aim and ambition—No. For no other calling would demand so many self-denials; in none would time for pleasure or amusement be less at your disposal; in none would your physical energies be more severely taxed; in none would your moral sensibilities be so painfully exercised. But, if to live for others' good; to relieve the distressed; to administer consolation to the anguish-stricken heart; to answer calls of charity and mercy; if to command the confidence and esteem of the wise and good, give you satisfaction—Yes. For no other business of life offers more frequent opportunities for blessing the suffering ones of our race: in none would you be led more directly to imitate the example of the Great Master who went about doing good.

The greatest achievements of the physician are not always those that are most loudly proclaimed to the public ear. Nor does the conscientious physician derive his highest gratification from listening to the tones of popular applause. His vocation seldom leads him into the noisy strife and busy excitements of life; but his mission is to the chamber of suffering. Look out upon the earth—the landscape is everywhere beautiful with flowers and fruit and grain; but over every mountain and valley, over city and plain and cottage, there rests a cloud—it is the shadow of the wings of Azrael and of his ten thousand inexorable ministers of death, and they are busy at their silent, ceaseless work. Do you not hear the wail of the mourners, breaking like sea-waves along the shore? There is sickness and pain and death everywhere across this beautiful earth. Wherever man flies, there these unrelenting foes follow. In all latitudes and climates, abroad and at home, the weary, panting fugitive still turns and struggles in vain to elude their pursuit, or to shake off their hateful companionship, and in his extremity he will appeal to you, who are to become the conservators of health and life, to ward off the blow of these destroyers, to turn back the march of these mortal foes—and your aid will be invoked to restore to the embrace of despairing friends one just vibrating between life and death. These are the physicians labors; such are his triumphs; and they afford him far more satisfaction a sweeter pleasure, than all the hollow acclamations of the populace.

I should wrong you, however, and misrepresent the profession, did I leave upon your minds the impression that these are the only rewards you are to expect in return for weary years spent in study in your lonely chamber—in the wards of the hospital, and in the mephitic atmosphere of the charnel-house; or for the labors, the sacrifices of personal comfort, and the anxieties inherent in the medical art. While I concede that ours is emphatically a benevolent calling, and that we should lend a willing ear to the call of pity and a helping hand to those in distress, yet the maxim drawn from holy writ, that "the laborer is worthy of his reward," has a binding force on the recipients of our sympathy and skill. I have just intimated that the medical profession does not open the way to princely fortune; nevertheless, I know of no occupation in life in which a competence may be more surely acquired than by the skillful physician; and never was there a time when the medical student was stimulated by so many incitements to perfect himself in the profession. In civil life the demand for competent physicians is as pressing as it has ever been. In the army and navy the need of educated surgeons is so great that promotions to the highest rank are sometimes reached before the second year. But please remember that the guarantees to success are scientific attainments and skill.

The labor before you is real. I invite you to no holiday pastime. You should see before you, and prepare for the earnest work of a useful life. A proper preparation requires not only the highest scientific attainments, but also the highest principles of honor. The noble men who have contributed most to elevate and advance our chosen profession have been the ornaments of society, as eminent for their virtues as for their scientific attainments and skill. Emulate their virtues, aspire to their attainments.

But I will detain you no longer. In the allotment of labor among your instructors it becomes my pleasant duty to present you our cordial greeting. With emotions, therefore, of pleasure and good will do I welcome you to these halls consecrated to the cultivation of medical science. For myself, Gentlemen, and in behalf of my colleagues, I would assure you that these are no formal, meaningless words. The intimate relations which are to exist between you, as members of the class, and the Faculty, as your instructors, shall bind us in sympathy and interest strong as fraternal ties. We meet those who have favored us before as old friends. Those who occupy these seats for the first time we welcome to the work which, although arduous, is not one of drudgery. I almost envy the pleasure in young and ardent minds of rising step by step in knowledge, and delighting in the wonders and beauties of the enlarging view, and "beholding the bright countenance of truth in the quiet and still air of delightful studies."

In the work which you have undertaken it needs no words of mine to stimulate you. Let the mission of our profession lead you on to improve the facilities offered you. Let the awfully momentous events now passing in the phenomena of the world be your monitors. You cannot, if you would, be indifferent spectators. While the crimson cloud of war rests over our beloved country—as if the angel was now emptying the vials of wrath—there is an imperative demand for your best efforts in her behalf; and, oh! let us devoutly hope, that the light now breaking in the distant horizon may not disappoint our earnest expectations—that the time is near when the curtain will be rolled up, though red with the best blood of your fathers and brothers, and that the dove with the olive branch may again spread her wings over a united, happy and prosperous people.

